# Clinical Significance of *Aspergillus* sp Found in Respiratory Fungal Cultures of ICU Patients

**DOI:** 10.1177/08850666251340043

**Published:** 2025-05-13

**Authors:** Katriina Pihlajamaa, Maija Halme, Miia Valkonen, Veli-Jukka Anttila

**Affiliations:** 1 Heart and Lung Center, 159841 Helsinki University Central Hospital, Helsinki, Finland; 2 3835 University of Helsinki, Helsinki, Finland; 3 Department of Perioperative, Intensive Care and Pain Medicine, Intensive Care Medicine, Helsinki University Central Hospital, Helsinki, Finland; 4 Inflammation Center, 159841 Helsinki University Central Hospital, Helsinki, Finland

**Keywords:** invasive pulmonary aspergillosis, fungal cultures, *aspergillus* colonization, diagnostic algorithm

## Abstract

**Background:** Invasive pulmonary aspergillosis (IPA) is a very severe manifestation of *Aspergillus* disease. Besides well-known risk groups of deeply neutropenic hematologic and solid organ transplant recipients other risk groups among patients treated in ICUs have been recognized. The prevalence of IPA among ICU-patients is not known and it is not known how well IPA is recognized in ICU-settings. The diagnosis of IPA is often difficult to make and non-invasive ways to diagnose IPA reliably are needed. **Objectives:** In this study we studied the clinical significance of *Aspergillus*-positive respiratory samples in ICU-patients. **Methods:** We retrospectively evaluated the ICU-patients (N = 205) who provided *Aspergillus*-positive respiratory samples in 2007-2020 and classified patients to groups of “colonization”, “putative IPA”, “proven IPA “, as in AspICU algorithm. Data were collected from laboratory registry and Helsinki University Hospital medical records. Underlying conditions, reasons leading to treatment in ICU, immunosuppression, known risk factors of IA in ICU, signs of infection, results of *Aspergillus*-specific laboratory testing, use of antifungal treatment, survival, and reason of death were assessed. **Results:** Majority of the findings (63%) were colonization, 11 (5%) patients had proven IPA, and “putative IPA” 59 (29%) of the patients. All patients with proven IPA died within one year, whereas mortality in putative and colonization groups was 39% and 33% respectively. Difference in mortality during one year between “colonization” and “putative IPA” groups was not statistically significant (p = .244), but when both “proven” and “putative” IPA were included, the difference was statistically significant, p = .019. Overall hospital mortality in the study group was 38%. Mortality in all the groups is higher than overall ICU-patient mortality of non-selected patients in Finland. **Conclusions:** The overall incidence of *Aspergillus*-findings in our ICUs was low. Isolation of *Aspergillus* in critically ill is associated with high mortality irrespective of invasion or colonization.

## Introduction

*Aspergillus* molds – commonly found in environment- can cause several types of infective and allergic diseases in humans, most common site of infection is respiratory tract. Pulmonary aspergillosis mostly affects those with immunosuppression or an underlying pulmonary disease. An extremely severe manifestation of *Aspergillus* disease is invasive pulmonary aspergillosis (IPA), which has long been well recognized, serious complication in classic risk groups of deeply neutropenic, mostly hematologic patients and stem cell transplantation or solid organ transplant recipients. However, there are also other risk groups of invasive aspergillosis (IA) patients treated in ICUs, and IA in ICU-settings has become a subject of interest. In ICU patients, *Aspergillus* most commonly enters through the lungs, from which it can disseminate.^1^ The incidence of invasive fungal infections (IFI) has been rising as increasing number of patients receive immunosuppressive treatment.^2^ Candida species account for roughly 80% of IFI in intensive care units,^2^ while *Aspergillus* species contribute to a range of 0.3% to 19%. Among *Aspergillus* infections, *Aspergillus* fumigatus is responsible for 95% of cases.^3^

Diagnosis of a disease caused by *Aspergillus* spp. is not easy, especially not among non-immunocompromised, but otherwise seriously ill patients. Diagnostic criteria for invasive aspergillosis have been developed for neutropenic patients.^4,5^ Unfortunately, the diagnostic tools useful in neutropenic patients such as detection of circulating aspergillus galactomannan (GM) antigen, are of little diagnostic value among nonneutropenic. To overcome the problem of differentiating between infection and colonization and to enhance the accurate identification of IA in ICU settings diagnostic algorithms have been proposed and validated.^6–9^

In this study, we aimed to classify patients in our own ICUs retrospectively according to the criteria published by Blot et al^8^ to find out how the potential infections and patients at risk are recognized, how antifungals are used and targeted, and what is the survival of patients with potential *Aspergillus*-infection.

## Material and Methods

All the *Aspergillus*-positive pulmonary fungal culture samples collected in 16 different ICUs treating adults in Helsinki University Central Hospital area between 2007-2020 comprise the material of this study. HusLab, which provides microbiological laboratory services for the area of approximately 1,65 million inhabitants in Helsinki University Hospital area in southern Finland, analyzed all the samples.

For fungal culture for specimens derived from respiratory tract HUSLAB uses Sabouraud plates with glucose (SabG) and maltose (SabM) and chromogenic plates. Plates are incubated in three different temperatures (+28, +37 and +45C) and grown for 14 days, growth is reported as soon as it is detected.

During the study time, samples derived from respiratory tract of 205 patients treated in ICUs revealed *Aspergillus*-growth in culture and were the subject of interest in this study.

In May and September 2022, the medical records in Helsinki University Hospital electronic patient record system (includes HusLab registry) of *Aspergillus*-positive ICU patients were comprehensively reviewed. Information on patients` underlying diseases, reasons leading to treatment in ICU, previous use of immunosuppressive medication, known risk factors of IA in ICU, signs of infection (clinical and radiological) during the ICU period, results of *Aspergillus*-specific laboratory testing when available, use of antifungal treatment, survival, and reason of death (when occurred) were addressed. Survival was monitored for a minimum of one year, including after hospital discharge.

The patients were retrospectively classified to groups of “colonization”, “putative IPA”, “proven IPA” as in AspICU algorithm by Blot et al (2012)^8^ extended with additional host risk factors,^10^ including acute viral infections (influenza and COVID-19). They are presented in Table 1. A few were classified as “chronic aspergillosis (CPA)” as stated by Denning et al^11^ As the material for this study came from registry of positive laboratory findings, all patients fulfilled one criterion for putative IPA: culture showing *Aspergillus* growth in respiratory specimen. Other criteria – at least one risk factor and clinical and radiological signs of pulmonary infection not otherwise explained – had to be fulfilled to be classified into category of “putative IPA”. Severe Covid-19 infection has recently been recognized as a potential risk factor for IA and has been added to the list of risk factors^12,13^ although cases in this material were few. Also, we used CDC /NHS definition of health care associated infections (HAI)^14^ the “proven IPA” group to identify health care associated infections.

**Table 1.** Criteria of Proven and Putative IPA According to Blot et al, Extended with Additional Host Risk Factors.

	Host risk factors	Clinical presentation	Microbiological or other laboratory evidence
Proven IA	Not required	Not required	PAD showing hyphae and tissue damage
Putative IA	At least one known risk factor:	And	
	systemic corticosteroid treatment prednisolone >20 mg/day	clinical and radiological signs of pulmonary infection otherwise unexplained	Microscopy or culture showing Aspergillus in lower respiratory tract specimen
	neutropenia <0,5		
	chronic pulmonary disease (COPD, bronchiectasis…)		and/ or GM >0,5 in serum or >0,8 in BAL
	decompensated liver disease		
	treatment with T-cell immunosuppressant		
	hematological malignancies / stem cell transplantation		
	solid organ transplantation		
	HIV		
	severe influenza		

The cases with histopathological confirmation of aspergillosis (N = 38) were used to calculate the sensitivity and specificity of the AspICU algorithm in this study.

SPSS Statistics version 27.01 was used for statistics analysis.

The research board of the Heart and Lung Centre of Helsinki University Hospital approved the study protocol. The ethics committee approval was not required because the subjects were not contacted during the study.

## Results

This study included all the *Aspergillus*-positive samples derived from patients in 16 ICUs between 2007-2020. During study time 205 patients had given *Aspergillus*-positive respiratory samples.

Between 2016-2020 *Aspergillus spp.*-growth in respiratory tract samples was detected in 104 (4,2%) of the samples taken from ICU-patients, and these samples were derived from 64 (varied between 8-15/year) patients, of whom 2 (3%) had proven IA and 21 (33%) putative IA.

Patient characteristics and risk factors for IA during the whole study time (2007-2020) are presented in Table 2.

**Table 2.** Patient Characteristics of 205 ICU-Patients in 2007-2020.

Age mean (range)	58,5 (19-85)
Male	126/205 (61%)
Invasive ventilation	180/205 (88%)
*Risk factors for IA*	
none	110/205 (54%)
Bone marrow transplant	0/205 (0%)
SOT^†^ recipients	29/205 (14%)
Previous‡ corticosteroid treatment	10/205 (4%)
Neutropenia <0,5	5/205 (2%)
chronic pulmonary disease ^§^	49/205 (24%)
decompensated liver disease	16/205 (8%)
T-cell influencing immunosuppressive medication^¶^	22/205 (11%)
HIV	0/205 (0%)
serious influenza	5/205 (2%)
serious Covid-19 infection	2/205 (1%)

^†^Solid organ transplant ‡long term dose >20 mg Prednisolone at least 3 weeks ^§^COPD, bronchiectasis.

^¶^ie calcineurin or mTOR-inhibitors.

Majority of fungal culture samples were tracheal suction samples or BAL samples as 180/205 (88%) patients were mechanically ventilated at the time of sample collection. Median time from admission to ICU to positive finding in fungal culture was 5 days (0-35).

Table 3 describes the groups according to retrospective classification.

**Table 3.** IA, Risk Factors.

	Colonization N = 130	Proven IPA N = 11	Putative IPA N = 59	Chronic aspergillosis N = 5
No risk factor	109 (84%)	0 (0%)	0 (0%)	1 (20%)
Systemic glucocorticoids	2 (2%)	5 (45%)	3 (4%)	0 (0%)
Neutropenia	1 (1%)	1 (9%)	3 (4%)	0 (0%)
Chronic pulmonary disease	11 (8%)	4(36%)	30 (51%)	4 (80%)
Decompensated liver disease	3 (2%)	5 (45%)	8 (14%)	0 (0%)
T-cell inhibitory medication	6 (5%)	8 (73%)	8 (14%)	0 (0%)
SOT	6 (5%)	3 (27%)	19 (32%)	1 (20%)
Serious Influenza	1 (1%)	1 (9%)	3 (4%)	0 (0%)
Covid-19	0 (0%)	0 (0%)	2 (3%)	0 (0%)
1 risk factor	12(9%)	4 (36%)	41 (69%)	3 (60%)
>1 risk factor	9 (7%)	7 (64%)	18 (31%)	1 (20%)

Majority of the findings (130/205,63%) classified as colonization. In autopsy was confirmed 11 (5%) of the patients to have proven IPA. Table 4 shows the characteristics of patients with proven IPA**.** In this group 7/11 cases (63,6%) were health care associated infections (HAI).

**Table 4.** Proven IPA-Patients.

Patient	Gender	Age	Reason of ICU treatment	Underlying diseases	HAI^†^/CA^§^	Antifungal medication	Days from first sample to death	Site of infection in autopsy	Reason of death
1	M	53	pulmonary infection	COPD and asthma	HAI	None	2	lungs	Invasive pulmonary aspergillosis
2	M	52	liver dysfunction	alcoholism, COPD	HAI	None	8	lungs	Liver cirrhosis, invasive pulmonary aspergillosis
3	F	58	pancreatitis, kidney dysfunction	previous lung transplantation	CA	micafungin	93	lungs	AAT1-deficiency, Pulmonary aspergillosis
4	M	40	liver dysfunction	alcoholism, hepatitis	CA	None	4	lungs, brain	Invasive aspergillosis
5	M	29	liver dysfunction	nephrotic syndrome (minimal change nephritis)	CA	None	1	lungs	Liver and kidney dysfunction
6	F	65	pneumonia	COPD	CA	None	1	lungs	Aspergillus pneumonia
7	F	52	hepatitis NAS	cryptogenic organizing pneumonia	HAI	None	2	lungs, liver, heart	Invasive aspergillosis
8	F	69	liver transplantation	autoimmune hepatitis	HAI	amphotericin B, voriconazole	25	lungs, brain, kidneys	Invasive aspergillosis
9	M	55	sepsis, pneumonia	MPO-vasculitis, kidney dysfunction (hemodialysis)	HAI	voriconazole	58	lungs	Aspergillus pneumonia, MPO-vasculitis
10	M	33	sepsis, cellulitis	GPA-vasculitis, obesity	HAI	amphotericin B, voriconazole	76	lungs, brain	vasculitis
11	F	62	liver transplantation	hepatitis NAS (acute)	HAI	amphotericin B, isavuconazole	13	lungs, pleural fluid	IPA, hepatitis NAS

^†^Health care associated infection. ^§^Community acquired infection.

Putative IPA -group included 59 (29%) patients. Five patients in this study had chronic aspergillosis diagnosed either previously or after treatment in ICU as in diagnostic criteria by Denning et al^11^

**Figure 1.**
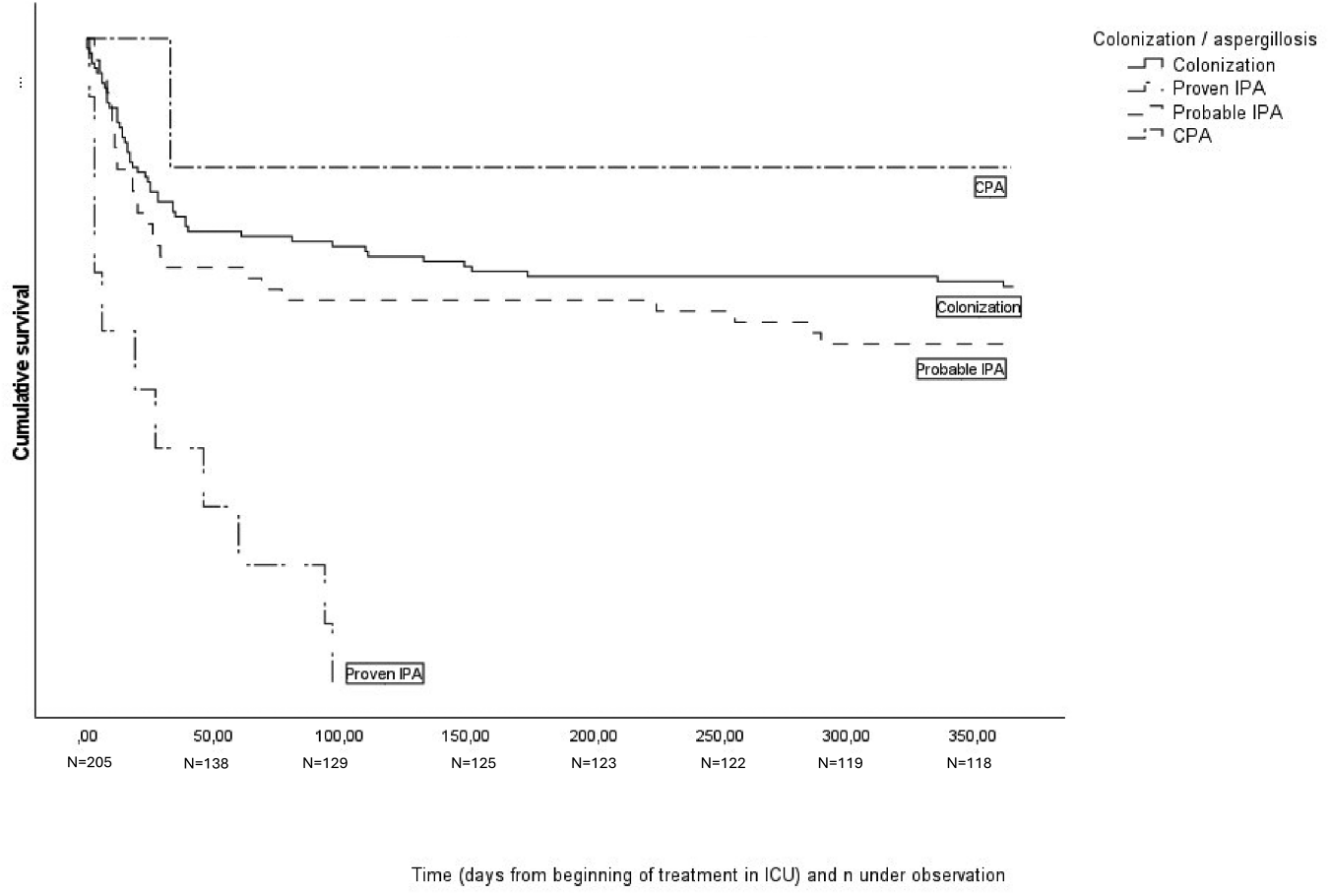
Survival from the day of admission to ICU.

The Kaplan-Meier curve in Figure 1 shows survival in one year starting from the day of admission to ICU. Within one year from the day of admission to ICU died 51/135 (38%) patients classified into category of colonization or chronic aspergillosis, and 28/59 (47%) of the patients in the category of “putative IPA”. All the “proven IPA” patients had died within a year, proven IPA was practically not possible to diagnose without autopsy because lung biopsy as an invasive method was too risky for mechanically ventilated patients. The median time of survival of “proven IPA” -patients was 19 days, the range was 1-96 days, one patient was discharged from hospital and later readmitted. Difference in mortality during one year between “colonization” and “putative IPA” groups was not statistically significant (p = .244), but when both “proven” and “putative” IPA were included, the difference compared to colonization group was statistically significant, p = .019. Hospital mortality in the study group was 77/205 (38%). In the “proven IPA” group hospital mortality was 91% (10/11), 39% (23/59) in “putative IPA” -group, and 33% (44/135) in combined colonization and chronic aspergillosis -groups.

Antifungal medication with efficacy against *Aspergillus*, either i.v. or per oral or both, received 46/205 (24%) patients (Table 5). Of the proven IPA cases 7/11 did not receive any fungal medication, most of them died within few days after admission to ICU and were diagnosed only after death. In the group of “putative IPA” 17/59 (29%) patients received some i.v. or p.o. antifungal medication. Also, in the colonization group 19/130 (15%) patients received medication i.v. or p.o. or both. Three patients received only inhaled antifungal treatment; these were routine treatments after lung transplantation.

**Table 5.** Antifungal Treatment.

	Colonization N = 130	Proven IPA (N = 11)	Putative IPA N = 59	Chronic aspergillosis N = 5	Died in hospital N = 77
Antifungal medication					
no i.v. / p.o. medication N = 162	111 (85,4%)	7 (63,6%)	42 (71,2%)	2 (40%)	65 (40,1%)
i.v. caspofungin N = 28	12 (9,2%)	0 (0%)	15 (25,4%)	1 (20%)	8 (28,6%)
i.v. amphotericin B N = 8	3 (2,3%)	3 (27,3%)	1 (1,7%)	1 (20%)	3 (37,5%)
p.o. voriconazole/posa/isavu N = 29	6 (4,6%)	4 (36,4%)	8 (13,6%)	2 (40%)	3 (10,3%)
inhaled amphotericin B N = 11	1 (0,8%)	0 (0%)	9 (15,3%)	1 (20%)	1 (9,0%)

Most frequently (n = 28) used i.v. medication was caspofungin, liposomal amphotericin B in eight cases. Voriconazole was the choice 16 times either as initial therapy or after i.v. treatment, isavuconazole in three cases and posaconazole in two. None of the patients received combination therapy as initial treatment, but it was used in five cases later during the treatment.

In our material pathology data (autopsy or in two cases explanted native lung) was available in 38 cases. In 11 cases autopsy confirmed invasive *Aspergillus* infection and in one case a chronic situation (CCPA). In the remaining 26 cases histopathological examination did not reveal aspergillosis. Of these 26 cases the clinical algorithm classified eight patients as putative IPA (false positives). The remaining 18 patients the algorithm correctly classified as colonization (true negatives). In this retrospective analysis of our small material, the sensitivity of the AspICU algorithm in cases with histopathological confirmation was 100% and specificity 69%.

## Discussion

Several risk factors for IA besides prolonged neutropenia and organ transplantation have been recognized: COPD in particularly with previous steroid treatment, liver failure and cirrhosis, serious influenza, and most recently Covid 19-infection.^2,15–18^ IPA has affected even previously healthy and immunocompetent individuals suffering from serious viral infections leading to treatment in ICU.^9^ According to some estimates, in ICU settings only 10%–15% of patients with IPA are neutropenic.^19^

Incidence of invasive aspergillosis in ICUs is unknown and naturally varies depending on the patients treated,^20^ also the environment can be a risk factor if concentration of *Aspergillus* spores in air is high as has been described in outbreaks during hospital construction.^21,22^ Geoclimatic environment probably influences the concentration of spores in air as well.^23,24^ It has been proposed that incidence of IA in ICU could be even 7%^25,26^ but due to difficulties in diagnostics, exact incidence remains unknown.

It is common to consider *Aspergillus* finding in a respiratory sample colonization or contamination, and therefore not initiate antifungal treatment. However, IPA can be one of the most frequently missed infections in ICUs,^27,28^and according to a recent systematic review of autopsy series even one third of missed pneumonia cases in ICU are IPA.^29^ Lack of suspicion of IA in non-neutropenic patients might lead to not taking fungal cultures at all, or to delayed initiation of treatment.^30^ Delays on appropriate treatment initiation worsen the outcome of the critically ill with other infections, and some studies suggest this also applies in IPA.^31,32^ On the other hand, it has been stated that 40%–70% of antifungal drug use is inappropriate.^3,33,34^

The prognosis of IPA is poor.^35,36^ However, the problem in diagnosis is how to get a sufficient histological sample representative of lung parenchyma from seriously ill and often mechanically ventilated ICU-patients. Therefore, there is a substantial need for noninvasive ways of diagnosing IPA reliably enough to target medication to the right patients without delays. Nevertheless, fungal culture results require several days and may not be promptly available for urgent clinical situations.

Creating diagnostic algorithm useful in clinical practice is challenging. In their observational validation study of the clinical algorithm where they included 524 ICU-patients with an Aspergillus-positive endotracheal aspirate culture,^8^ Blot et al resulted in sensitivity of 92% and specificity of 61% for their algorithm based on pathology data of 115 patients. In our study, the AspICU algorithm would have found all the 11 patients who had proven IPA and there would not have been false negatives. Sensitivity in retrospective analysis was 100% and specificity 69%. Aspergillus GM-antigen level when detected in BAL-fluid appears to be more informative than circulating *Aspergillus* antigen in blood in non-neutropenic patients. Beta-D-Glucan (BDG) -testing is another biomarker of fungal diseases, but its usefulness in diagnosing IA in nonneutropenic is limited due to low specificity.^37^ In algorithm BmAspICU biomarkers are included in addition to the criteria presented in AspICU.^38^ The use of BmAspICU -approach was not possible here due to a limited number of biomarker results, additionally BDG-testing was not available in our laboratory.

Outbreaks of *Aspergillus* disease in ICUs have been described.^22^ Hospital wards and ICUs dedicated to treating severely neutropenic patients have implemented High Efficiency Particulate Air (HEPA) filter systems. This measure aims to mitigate the risk of infections stemming from ICU contamination and to curtail outbreaks associated with ubiquitous fungi. After introduction of HEPA systems, the *Aspergillus* spore load in the air and incidence of invasive aspergillosis among hematologic patients has decreased.^39^ In our material there were no suspected hospital outbreaks.

The overall incidence of *Aspergillus*-findings in our ICUs was low. The fungal culture samples derived from ICU-patients were positive for *Aspergillus* in 4,2% in 2016-2020. It is routine to collect samples for fungal cultures from mechanically ventilated patients who have undergone solid organ transplantation (lung or heart) during their ICU treatment post-transplantation, and many of the fungal cultures taken in ICUs were these samples. The low overall incidence of *Aspergillus*-findings in this study could partly be due to geoclimatic influences. It is also noteworthy that even though all *Aspergillus*-findings were included, hematologic patients were almost absent from this material and none of the proven IPA -cases were hematologic patients. Since the late 1990s, our ICUs treating neutropenic patients have used HEPA systems for incoming air.

Patients treated in ICU often suffer of several conditions which potentially can lead to death. The group of ICU-patients is very heterogenous, and mortality is highly dependent on the presence and severity of organ system dysfunctions and failures. In this study hospital mortality was higher than in a large Finnish study of non-selected ICU patients – 38% in our study and 10,7% in the study of Pölkki et al.^40^ This applies also in *Aspergillus* colonization group, where hospital mortality was 33%. However, Helsinki University Hospital is the only tertiary transplantation center in Finland and the patient population may not be representative for most ICUs in the country.

Limitations of this study include its retrospective nature. Also, it is possible or even likely that all cases of IPA in our ICUs are not included in this study. This study includes only those who had a positive *Aspergillus*-finding in culture – not the patients whose cultures were negative or who did not provide samples for fungal culture at all. Also, IPA can occur in the absence of positive respiratory cultures.^41^ Also, retrospective decision if clinical and radiological signs of infection could be explained otherwise and caused by another infection, is difficult to make. Taking the fungal cultures and resulting in a positive *Aspergillus*-finding did not always lead to initiation of treatment with antifungals even though risk factors were present. In the group “putative IPA” many patients did not suffer from IPA and survived without any antifungal treatment. However, it is likely that in this category there were also true IPA-cases. Since the diagnosis of “proven IPA” in this material was based on autopsy findings, it inherently introduces a bias towards higher mortality in this group. In clinical practice, it would also be important to distinguish whether a positive *Aspergillus* finding indicates a proven or putative infection, or merely colonization.

In conclusion, hospital mortality was high in all groups when *Aspergillus* was isolated in critically ill patients, regardless of whether it was identified as colonization or invasion.
